# Dynamic regulatory mechanism of cholesterol 25-hydroxylase-mediated antiviral response in RNA virus infections

**DOI:** 10.3389/fcimb.2026.1790475

**Published:** 2026-07-07

**Authors:** Zhengqi Liang, Simiao Xing, Yujia Chen, Miaoxin Liu, Suhua Chang, Yuchen Cai, Qing Xiong, Yao Xu, Jinbiao Liu

**Affiliations:** 1 National “111” Center for Cellular Regulation and Molecular Pharmaceutics, Key Laboratory of Fermentation Engineering (Ministry of Education), Hubei Provincial Cooperative Innovation Center of Industrial Fermentation, School of Life and Health Sciences, Hubei University of Technology, Wuhan, Hubei, China; 2 Institute of Biology and Medicine, College of Life Science and Health, Wuhan University of Science and Technology, Wuhan, Hubei, China

**Keywords:** antiviral response, CH25H, cholesterol, lipid metabolism, regulatory mechanisms, RNA virus evasion

## Abstract

Due to their high mutation rates and adaptability, RNA viruses pose a persistent threat to public health. Cholesterol-25-hydroxylase (CH25H), an interferon-stimulated gene (ISG), produces 25-hydroxycholesterol (25HC), which plays a pivotal role in host defense against RNA virus infections. However, infection outcomes are determined by the dynamic interplay between viral infection and host immune defense, including cholesterol metabolism mediated by CH25H/25HC axis. This review aims to comprehensively elucidate the mechanisms underlying the interaction between CH25H/25HC and RNA viruses. We summarize recent advances in understanding the antiviral mechanisms of CH25H/25HC against RNA viruses, highlighting the central role of CH25H and its metabolite 25HC in inhibiting viral replication and regulating immune cell function. Furthermore, we discuss how RNA viruses evade host immune surveillance through strategies such as gene mutation, suppression of immune pathways, and interference with CH25H expression or function. Moreover, we emphasize endolysosomal cholesterol homeostasis as a host determinant of RNA virus entry, endosomal escape, trafficking, and replication, and discuss cholesterol-modulating host-directed therapies as complementary strategies to direct-acting antivirals. The findings indicate that dynamic regulation of CH25H and its metabolite 25HC is crucial for maintaining a balanced innate immune response against RNA viruses. This review comprehensively elucidated the dynamic molecular interactions between CH25H and RNA viruses, integrating recent research advances with a focus on molecular regulatory mechanisms. By synthesizing these findings, this review provided a mechanistic framework for understanding host–virus conflicts centered on cholesterol metabolism and proposed potential therapeutic strategies targeting this axis.

## 1 Introduction

Viruses hijack cellular key factors in lipid metabolism and the membrane to support the life cycle in different steps of replication (Omasta and Tomaskova, 2022). The interaction between viruses and lipids has always been the cutting edge of research. RNA viruses attracted great attention due to their high mutation rates and broad host ranges. RNA virus infections pose a significant challenge to global public health, with the disease burden continuing to intensify over the past two decades (Debat and Bejerman, 2025). Influenza viruses result in 3 to 5 million cases of severe illness and 290, 000 to 650, 000 deaths worldwide (World Health Organ, 2025a). Influenza virus is an enveloped virus that relies heavily on host cellular metabolic machinery, particularly the lipid metabolic system, to facilitate key steps in its life cycle—ranging from initial interactions with host cells and membrane fusion to nuclear import and export, as well as the coordination of viral particle assembly and budding (Nayak et al., 2009; Heaton and Randall, 2011; Zhou et al., 2021). The disease caused by severe acute respiratory syndrome coronavirus 2 (SARS-CoV-2) pandemic has further highlighted the threat of RNA viruses. As of May 27, 2020, there were 5, 715, 077 confirmed cases of SARS-CoV-2 globally, including 352, 912 deaths, causing profound impacts on global economies and societal order (Feng et al., 2020). SARS-CoV-2 infection leads to alterations in lipid profiles (Wei et al., 2020; Kowalska et al., 2022), can affect multiple organs throughout the body via the angiotensin-converting enzyme 2 (ACE2) receptor, and induces dysregulation of lipid metabolism (Gheblawi et al., 2020; Munawar et al., 2024). Hepatitis C virus, a representative chronic RNA viral infection, affects approximately 58 million people worldwide and is responsible for around 290, 000 deaths annually due to be infected with HCV, an estimated 1.5 million new infections occur each year (World Health Organ, 2021). The hepatitis C virus (HCV) exists as lipid-rich particles, invades hepatocytes via lipoprotein receptors, and upregulates host lipid synthesis while inhibiting its degradation, leading to substantial lipid accumulation in hepatocytes and reduced cholesterol levels in the circulating blood (Elgretli et al., 2023). Furthermore, the periodic outbreaks of emerging RNA viruses such as dengue, Zika, and Ebola viruses exacerbate the global disease burden (Christie et al., 2023; Parveen et al., 2023; Izudi and Bajunirwe, 2024). Approximately 390 million people are infected with the dengue virus annually (Dong et al., 2025), of whom approximately 500, 000 progress to severe disease and approximately 20, 000 die due to hemorrhagic complications (Khetarpal and Khanna, 2016). During the stages of viral entry, replication, and assembly, DENV selectively exploits lipid metabolism-related enzymes to modulate the host cellular metabolic system, thereby creating a metabolic state conducive to the completion of the viral replication cycle (Xie et al., 2025). To date, a total of 92 countries and territories have reported evidence of mosquito-transmitted Zika virus infection (World Health Organ, 2025b). Zika virus replication is similarly dependent on lipid and cholesterol metabolism. During replication, the virus generates a large amount of endoplasmic reticulum-derived lipid droplets to support membrane synthesis or provide energy for replication (Cortese et al., 2017; Leier et al., 2020; Stoyanova et al., 2023). These data highlight the facts that RNA virus infection is worldwide spread and poses a huge threat to human health underscoring the necessity for in-depth study of viral infection and lipid metabolism. The pervasive reliance of diverse RNA viruses on host lipid metabolism and membrane dynamics reveals a conserved vulnerability in their life cycles. This virus-host lipid metabolic interplay not only underpins viral pathogenesis and global disease burden but also identifies lipid-centered pathways as promising broad-spectrum antiviral targets.

Cellular lipids can be categorized into membrane lipids (including phospholipids, sphingolipids, and glycolipids), cholesterol, steroids, triacylglycerols (TAGs), fatty acids, and eicosanoids (Fahy et al., 2011). Viruses interact with host cell lipids through diverse mechanisms, and accumulating evidence indicates that lipid metabolism and lipid droplets (LDs) are involved in multiple stages of RNA virus infection, including viral replication and assembly (Konan and Sanchez-Felipe, 2014; Farías et al., 2024). Inhibition of enzymes involved in cellular fatty acid biosynthesis or cellular lipases responsible for lipid droplet degradation impairs the function of viroplasms in rotaviruses or replication compartments in other RNA viruses, thereby reducing the production of infectious progeny virions (Desselberger, 2024). Phosphatidylinositol (PI) and its mono- and bis-phosphate derivatives play critical roles in the formation of replication complexes in enteroviruses and other positive-sense single-stranded RNA viruses (Zhang et al., 2019b). In coronaviruses, particularly SARS-CoV-2 which causes COVID-19, ceramide plays a central role in viral entry into host cells (Cesar-Silva et al., 2023). The NS5A protein of hepatitis C virus (HCV) binds to the surface of lipid droplets (LDs) and interacts with cellular cyclophilin A (CypA), participating in viral RNA replication in HCV-infected cells (Madan et al., 2014). Metabolites derived from lipid metabolism are directly involved in immune cell activation and the initiation of inflammatory signaling pathways (Andersen, 2022). Upon recognition of pathogens by innate immune cells such as macrophages and dendritic cells, lipid rafts in the plasma membrane rapidly reorganize, becoming enriched in cholesterol and sphingolipids to form activation platforms for pattern recognition receptors, including Toll-like receptors (TLRs) and NOD-like receptors (NLRs) (Triantafilou et al., 2011). The lipid metabolic intermediate sphingosine-1-phosphate acts as a signaling molecule, regulating immune cell migration and inflammation site aggregation through G-protein coupled receptors (Sun et al., 2024). These processes highlight that lipids are not only structural components of cells but also key messengers driving innate immune responses (Cabral, 2005). Upon immune activation, inflammatory factors directly alter the activity of metabolic enzymes and lipid biosynthesis pathways (York et al., 2015). Collectively, cellular lipids play a dynamic dual role in viral infection and host immunity: on one hand, viruses hijack lipid metabolism, exploit lipid droplets and specific lipid classes to facilitate their entry, replication, and assembly; on the other hand, lipid metabolites directly participate in the activation of innate immune cells, initiation of inflammatory signaling, and regulation of immune feedback, thereby forming a lipid-mediated interactive network between the virus and host immunity.

As a key component of lipids, cholesterol is of great involvement in cell homeostasis (Kulig et al., 2016), and immune responses (Fessler, 2016). CH25H is a multi-transmembrane endoplasmic reticulum (ER)-associated enzyme that catalyzes the conversion of cholesterol to 25HC. The resulting 25HC acts as a signaling molecule that effectively inhibits sterol biosynthesis, primarily by suppressing the proteolytic activation and function of sterol regulatory element-binding proteins (SREBPs), thereby reducing intracellular cholesterol accumulation (Adams et al., 2004; Radhakrishnan et al., 2007; Zhao et al., 2020; Saito et al., 2023). CH25H and 25HC have been proven to play important roles in lipid metabolism and immune responses (Zhao et al., 2020). Meanwhile, CH25H demonstrates promising potential in antiviral infection (Yousefi et al., 2023). As an interferon-stimulated gene, CH25H is induced in response to viral detection, primarily by activating pattern recognition receptors (PRRs) (Zhao et al., 2020). As an endogenous antiviral molecule, 25HC has been demonstrated to suppress the replication of multiple RNA viruses, including vesicular stomatitis virus (VSV) (Liu et al., 2013), human immunodeficiency virus (HIV) (Moog et al., 1998), hepatitis C virus (HCV) (Anggakusuma et al., 2015), Ebola virus (EBOV) (Liu et al., 2013), porcine reproductive and respiratory syndrome virus (PRRSV) (Song et al., 2017), etc. Notably, CH25H not only plays a critical role in antiviral immunity but also significantly contributes to lipid metabolism and host defense mechanisms (Yousefi et al., 2023). Therefore, the study of CH25H will provide crucial insights into host antiviral immune mechanisms and offer valuable implications for developing novel therapeutic strategies against viral diseases and malignancies.

In addition to global cholesterol biosynthesis and plasma membrane cholesterol accessibility, endolysosomal cholesterol homeostasis has emerged as a critical determinant of RNA virus infection (Glitscher and Hildt, 2021; Post et al., 2025). Many enveloped RNA viruses enter host cells through endocytic routes and require the coordination of endosomal acidification, cholesterol trafficking, membrane remodeling, and fusion machinery to complete viral uncoating and cytoplasmic release (Glitscher and Hildt, 2021; Post et al., 2025). Intervention of cholesterol transport within late endosomes and lysosomes can therefore interfere with viral entry at a conserved host-dependent step. This concept is particularly relevant to influenza A virus (IAV), SARS-CoV-2, and Ebola virus (EBOV). Pharmacological disruption of endolysosomal cholesterol balance, for example, by itraconazole, fluoxetine, or other functional inhibitors of acid sphingomyelinase, has been shown to induce cholesterol accumulation and impair endolysosomal acidification, thereby restricting viral entry or early post-entry events in these viruses (Kuhnl et al., 2018; Schloer et al., 2019; Schloer et al., 2020a; Kummer et al., 2022; Schloer et al., 2022). These findings highlight the antiviral importance of CH25H/25HC mediated cholesterol metabolism and suggest that the endolysosomal cholesterol network could serve as a broader host−directed target against emerging RNA viruses (Glitscher and Hildt, 2021; Post et al., 2025).

Although previous reviews have discussed the antiviral functions of CH25H in the context of lipid metabolism (Yousefi et al., 2023; Zhong et al., 2023), a comprehensive summary of how clinically important RNA viruses-such as Ebola virus (EBOV), Encephalomyocarditis virus (EMCV), Human Rhinovirus (HRhV), Human Rotavirus (HRoV), and Zika virus, targeting CH25H through complex molecular mechanisms to achieve immune evasion remains insufficiently elucidated. This is particularly true for understanding the dynamic regulatory patterns underlying this process during human and animal RNA virus infections. The primary objective of this review is to comprehensively dissect how RNA viruses dynamically adjust their evasion strategies to subvert CH25H-mediated host defense. To this end, we will focus on delineating the multi-layered strategies employed by viruses, including direct inhibition of CH25H transcription, interference with upstream immune signaling pathways, remodeling of host lipid metabolism to alter the functional milieu of 25HC, and potential involvement of RNA interference mechanisms. More importantly, from a dynamically integrated perspective, this review aims to reveal how these inhibitory mechanisms act in concert at different stages of viral infection. This review will fill the current knowledge gap in the field and provide a comprehensive insight of how RNA viruses evade host innate immune defenses against CH25H and its metabolite 25HC.

## 2 Biological functions of CH25H

**Figure 1:**
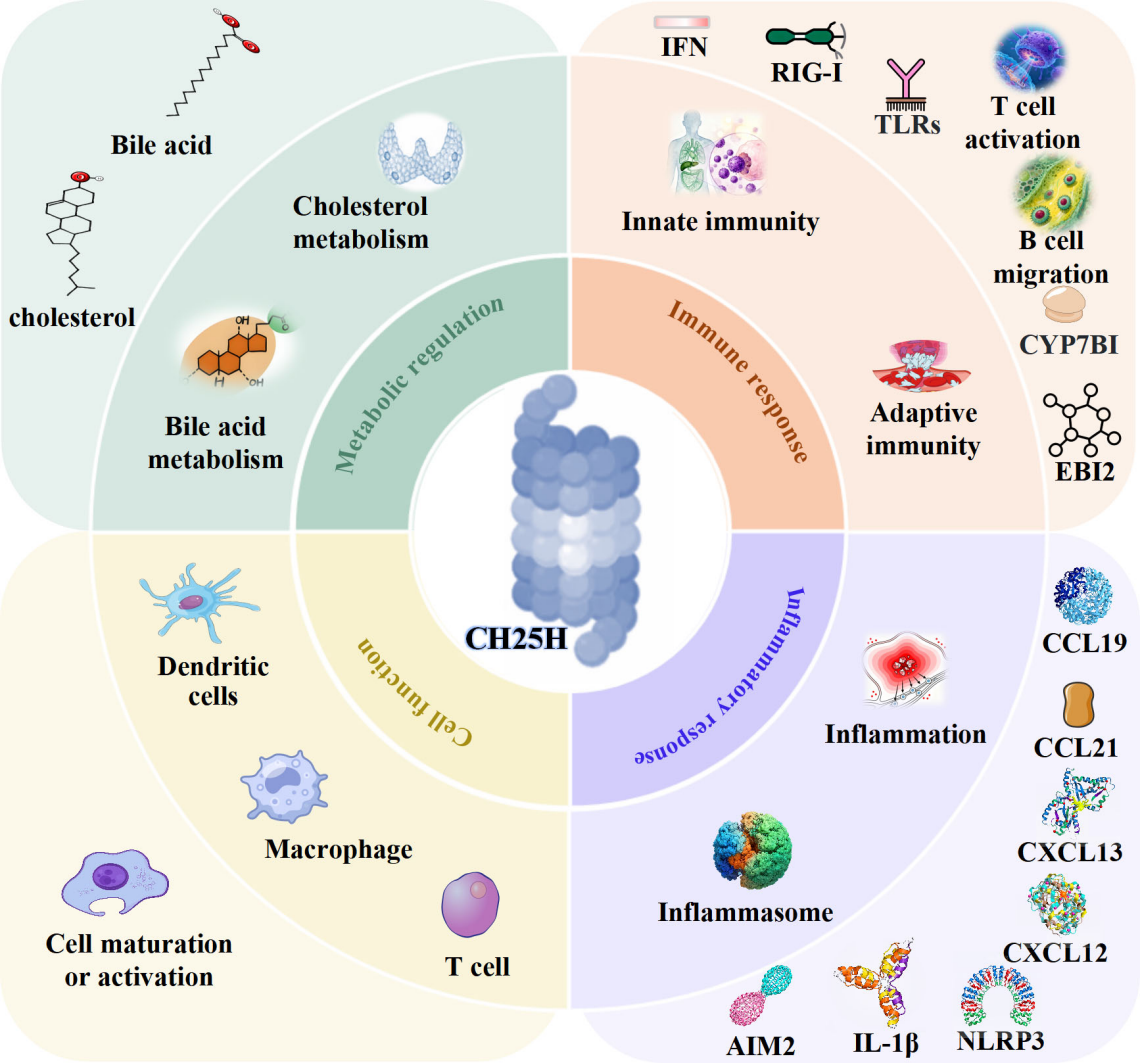
Biological functions of CH25H. CH25H exerts central regulatory functions across multiple physiological and pathological levels through its catalytic production of 25HC. At the metabolic level, 25HC acts as an endogenous ligand of LXR, dynamically regulating cholesterol homeostasis by activating cholesterol efflux and inhibiting SREBP-mediated synthesis. At the immune level, CH25H/25HC modulates the SREBP and LXR pathways to suppress inflammation and enhance anti-pathogen defense in innate immunity, while in adaptive immunity, it regulates cell migration, antibody production, and T cell polarization. At the inflammatory level, 25HC directly exerts negative regulatory effects on inflammatory responses, amplifies antiviral signals through a positive feedback loop with interferon, and suppresses excessive inflammation via multiple pathways under conditions of viral infection. These interconnected functions enable the host to establish a dynamic, multi-layered defense and homeostasis network through precise regulation of CH25H expression, thereby responding to diverse challenges such as viral infection and metabolic imbalance.

Cholesterol constitutes approximately 20% of lipids in the plasma membrane and serves as a pivotal regulator involved in cellular homeostasis, including lipid metabolism, cell function, and immune responses. As a key cholesterol-metabolizing enzyme, CH25H converts cholesterol into 25HC and plays critical roles in cellular homeostasis mediated by cholesterol and 25HC (**Figure 1**).

### 2.1 Cholesterol metabolism and regulation

Cholesterol homeostasis, which is critical for cell survival, is maintained via a negative feedback loop. Cholesterol biosynthesis generates key intermediate compounds, including isoprenoids and oxysterols, which play essential roles in various cellular processes. Cellular cholesterol homeostasis is primarily governed by two key transcriptional regulators: sterol regulatory element-binding proteins (SREBPs) and liver X receptors (LXRs) (Zhao et al., 2020). Under low intracellular cholesterol conditions, SREBPs form a complex with the SREBP cleavage-activating protein (SCAP) and translocate from the endoplasmic reticulum to the Golgi apparatus, where they are proteolytically activated by site-1 protease (S1P) and site-2 protease (S2P). The resulting mature transcription factors then enter the nucleus to initiate the expression of genes involved in the mevalonate pathway, including the rate-limiting enzyme HMG-CoA reductase, thereby promoting cholesterol synthesis (Brown and Goldstein, 1997). In contrast, LXRs are nuclear receptor transcription factors that are activated when intracellular cholesterol levels are abundant, subsequently inducing the expression of genes involved in cholesterol absorption, degradation, transport, and excretion (Zelcer and Tontonoz, 2006).

CH25H, which belongs to the cholesterol-metabolizing enzyme family and consists of 298 amino acids in human cells, is primarily localized in the ER and Golgi apparatus. The primary function of CH25H resides in its catalytic activity, which mediates the hydroxylation of cholesterol to 25HC via its oxidase activity (Zhao et al., 2020). 25HC, an oxysterol produced by CH25H catalysis, serves as a central molecule linking innate immunity and metabolic regulation and plays a significant role in cholesterol homeostasis (Brown and Goldstein, 2009; Wong et al., 2020). 25HC can activate LXRs to initiate a positive regulatory loop, promoting the expression of downstream target genes, including CH25H itself. As an endogenous ligand for LXRs, 25HC also interacts with SREBPs to modulate cholesterol biosynthetic and uptake pathways (Goldstein et al., 2006). When intracellular cholesterol is abundant, endogenous ligands such as 25HC activate LXR signaling, thereby inducing genes involved in cholesterol absorption, degradation, transport, and excretion to maintain cholesterol homeostasis (Lehmann et al., 1997). Under conditions of cholesterol excess, 25HC binds to membrane-spanning ER protein Insulin-induced gene 2 (INSIG2) proteins, which stabilize the interaction between INSIG and SCAP, thereby retaining the SREBP-SCAP complex within the ER. Consequently, SREBPs remain inactive, thereby inhibiting cholesterol synthesis (Radhakrishnan et al., 2004). In Chinese hamster ovary (CHO) cells, 25HC inhibits the activity of 3-hydroxy-3-methylglutaryl-CoA reductase by inactivating SREBP2, thereby reducing cholesterol biosynthesis (Adams et al., 2004). In macrophages, 25HC acts as an LXR ligand to regulate cholesterol and fatty acid synthesis pathways by upregulating the expression of genes such as SREBP1 (Ma et al., 2008). In mouse Schwann IMS32 cells, 25HC suppresses the processing of SREBP1, downregulating the key antioxidant glutathione peroxidase 4 (GPX4) at both transcriptional and translational levels. Concurrently, it upregulates the expression of the pro-lipid peroxidation enzymes NADH-cytochrome b5 reductase 1 (CYB5R1) and NADPH-cytochrome P450 reductase (POR), and increases the expression of glutathione-specific gamma-glutamylcyclotransferase 1 (CHAC1) to deplete glutathione. Collectively, these effects lead to redox imbalance and lipid peroxidation in the cells (Urano et al., 2025). These different cell models have revealed that mechanisms, including the regulation of cholesterol homeostasis, lipid metabolism, and oxidative stress, collectively delineate the core framework of 25HC action. Given the high sequence and functional conservation of CH25H and its product 25HC in vertebrates, the above mechanisms likely represent a ubiquitous and conserved regulatory logic operating in diverse cell types and even in overall host defense, rather than being restricted to specific cell types. In addition to suppressing cholesterol biosynthesis, 25HC also promotes the conversion of cholesterol to bile acids and the efflux of intracellular cholesterol in an LXR-dependent manner (Zhao et al., 2020). As a LXR ligand, 25HC induces the expression of cholesterol efflux transporters ATP-binding cassette transporter A1 (ABCA1) and ATP-binding cassette transporter G1 (ABCG1), as well as cholesterol sulfotransferase 2B1b (SULT2B1b). It also upregulates Apolipoprotein E (ApoE) and cytochrome P450 family 7 subfamily A member 1 (CYP7A1), thereby promoting cholesterol transport and clearance to maintain cholesterol homeostasis (Choi and Finlay, 2020). Additionally, 25HC promotes interferon-γ expression in an LXR-dependent manner; interferon-γ, in turn, upregulates CH25H synthesis (Liu et al., 2018b), further enhancing 25HC production and establishing a positive feedback loop (Liu et al., 2018a). Cholesterol esterification is mediated by acetyl-CoA acetyltransferases (ACAT), which alleviate free cholesterol toxicity and facilitate storage in lipid droplets (Olzmann and Carvalho, 2019; Mathiowetz and Olzmann, 2024). By enhancing ACAT activity, 25HC drives cholesteryl ester storage within lipid droplets (LDs), promoting the redistribution of accessible cholesterol at the plasma membrane and thereby restricting viral infection (Wang et al., 2020). In hepatocytes, 25HC-activated LXRα induces cholesterol 7α-hydroxylase (CYP7A1), the rate-limiting enzyme in the classical bile acid synthesis pathway, thereby facilitating the conversion of cholesterol into bile acids (Wang and Tontonoz, 2018). Studies using LLC-PK1 cells have shown that 25HC evidently inhibits infection by Porcine deltacoronavirus (PDCoV), with its effects primarily targeting the early and middle stages following viral entry. 25HC modulates cholesterol metabolism dysregulation induced by PDCoV infection through Transforming growth factor β1 (TGF-β1) and promotes the accumulation of interferon-related lipid droplets (Zhang et al., 2022). Within this regulatory framework, CH25H and 25HC are the two key regulators that maintain cholesterol homeostasis by inhibiting the SREBP pathway (which promotes cholesterol synthesis) and activating the LXR pathway (which promotes cholesterol efflux).

### 2.2 Immune regulation

CH25H and 25HC not only play critical roles in regulating cholesterol metabolism but also function as key modulators of innate and adaptive immunity through the SREBP and LXR pathways (Spann and Glass, 2013).

In innate immunity, the CH25H gene is an interferon-stimulated gene (ISG) whose expression is tightly regulated by innate immune responses (Mogensen, 2009; Zhang et al., 2018). Recognition of viral pathogen-associated molecular patterns (PAMPs) by pattern recognition receptors (such as TLRs and RLRs) in immune cells activates interferon signaling pathways, thereby inducing CH25H expression (Jensen and Thomsen, 2012). Viral infection and TLR ligands significantly upregulate CH25H expression in animal cells, a process dependent on the interferon signaling pathway (IFNR/JAK/STAT1) (Song et al., 2017; Wu et al., 2018b; Xie et al., 2019). Activation of Toll-like receptors (TLRs) increases CH25H expression, leading to enhanced production of 25HC (Park and Scott, 2010). 25HC, on one hand, inhibits IL-1 transcription and inflammasome activation by antagonizing SREBP processing (Reboldi et al., 2014); on the other hand, it serves as an endogenous ligand for LXR to enhance anti-pathogen responses (Liu et al., 2018b).

In adaptive immunity, 25HC and its metabolite 7α-25HC are ligands for the G protein-coupled receptor EBI2 (GPR183), participating in the regulation of migration and positioning of immune cells such as B cells and dendritic cells (Emgård et al., 2018). 25HC inhibits the SREBP2 pathway, suppresses IgA responses (Trindade et al., 2021), and influences the conversion of CD4+ T cells from pro-inflammatory to anti-inflammatory phenotypes by modulating cholesterol flux, selectively inhibiting the secretion of cytokines such as IL-2 and TNF-α (Wu et al., 2018b; Perucha et al., 2019). A recent study demonstrated that the CH25H/25HC-GPR155-mTORC1-AMPKα-STAT6 axis constitutes a critical immunometabolic regulatory pathway that receives immunosuppressive signals from the tumor microenvironment and, through metabolic and signaling pathway modulation, “locks” macrophages into an immunosuppressive state, thereby inhibiting the antitumor function of CD8+ T cells (Xiao et al., 2024). These mechanisms share mechanistic commonalities or engage in cross-talk with potential pathways of immune regulation in antiviral responses.

### 2.3 Regulatory mechanisms of CH25H expression

The expression of CH25H is finely regulated via multiple mechanisms, which are primarily influenced by both external stimuli and internal pathways (**Figure 1**). CH25H expression is upregulated during infections, inflammation responses, and immune responses. During viral infections and bacterial invasions, host cells recognize pathogens TLRs and RLRs, activating downstream type I interferon (IFN) pathways, which in turn induce CH25H expression (Jensen and Thomsen, 2012; Liu et al., 2018a). Studies have shown that multiple Toll-like receptors (TLRs) recognize specific molecular components of different viruses to initiate antiviral immune responses. Specifically, TLR7 and TLR8 primarily recognize viral single-stranded RNA (ssRNA). TLR7 is known to be activated by ssRNA from various viruses, including Influenza A virus (Diebold et al., 2004), Vesicular stomatitis virus (Lund et al., 2004), Human immunodeficiency virus (HIV) (Hardy et al., 2007), and Dengue virus (Wang et al., 2006). TLR8 has also been reported to recognize ssRNA from HIV (Han et al., 2012). TLR3 specifically recognizes viral double-stranded RNA (dsRNA), and viruses such as Reoviridae (Harada et al., 2007), Respiratory syncytial virus (Huang et al., 2008), and Influenza A virus (Guillot et al., 2005) are sensed by TLR3 through their dsRNA. TLR2 and TLR4 primarily recognize viral proteins. For instance, TLR2 recognizes the hemagglutinin (HA) of Measles virus (Bieback et al., 2002) as well as the core protein and NS3 protein of Hepatitis C virus (Dolganiuc et al., 2004); TLR4 recognizes the fusion protein of Respiratory syncytial virus (Kurt-Jones et al., 2000) and the envelope protein of Mouse mammary tumor virus (Rassa et al., 2002). The upregulation of CH25H is an early response of host cells to pathogen invasion, thereby facilitating the initiation of an effective immune defense. In addition to IFNs, transcription factors also play an imperative role in the regulation of CH25H. Tumor Necrosis Factor-alpha (TNF) induces the expression of CH25H through activation of the NFκB and MAPK signaling pathways (Pokharel et al., 2021). Specifically, upon TNF stimulation, Activating Transcription Factor 2 (ATF2), a member of the Activator Protein-1 (AP-1) family downstream of the MAPK pathway, directly binds to the promoter region of the CH25H gene, thereby driving its transcription (Pokharel et al., 2021). Lipopolysaccharide (LPS) has been shown to elevate CH25H levels in macrophages from both mice and human volunteers. This increase in CH25H expression is independent of myeloid differentiation factor 88 (MyD88) signaling but rather dependent on Toll-like receptor 4 (TLR4) signaling (Diczfalusy et al., 2009). The expression level of CH25H undergoes significant changes after viral infection. This process is primarily mediated by the activation of interferon signaling and other inflammatory pathways. Upon viral invasion, PRRs within host cells, as well as Toll-like receptors 3 (TLR3) and 7 (TLR7) on the cell surface, recognize viral RNA and activate several signaling pathways (Carty et al., 2021; Chen et al., 2025). This leads to the production of type I interferon-β (IFN-β). IFN-β subsequently binds to the heterodimeric interferon receptor (IFNR) on the cell surface, thereby activating the JAK-STAT signaling pathway (Park and Scott, 2010). This results in the phosphorylation of STAT1 and STAT2, leading to the formation of a heterodimer between them. In conjunction with interferon regulatory factor 9 (IRF9), they form the interferon-stimulated gene factor 3 (ISGF3) complex, which enters the nucleus and binds to specific interferon-stimulated response elements (ISREs), thereby promoting the transcription of CH25H and other antiviral genes (Mahjoor et al., 2023; Muhammad et al., 2025).

Dendritic cells, macrophages, and T cells are the primary cell types that express CH25H, and the maturation, activation status, and functional state of these cells directly influence CH25H expression levels (Park and Scott, 2010; Blanc et al., 2013). Studies have shown that the promoter region of CH25H may be regulated via epigenetic mechanisms by DNA methylation (Tsujioka et al., 2015). Demethylation of the CH25H promoter region promote CH25H transcriptional activity, thereby enhancing the sensitivity of antiviral immune responses (Tsujioka et al., 2015). 25HC itself can activate CH25H expression, thereby establishing a positive feedback loop, an effect that is dependent on LXRs (Liu et al., 2018a). Inflammatory cytokines, including interleukin-1β (IL-1β), tumor necrosis factor-α (TNFα), and IL-6, also promote CH25H expression in virally infected human macrophages via the STAT1 transcription factor (Magoro et al., 2019). Activating transcription factor 3 (ATF3) has been reported to function as a negative regulator of the Ch25h gene by directly binding to its promoter and epigenetically repressing its expression (Gold et al., 2012).

**Figure 2:**
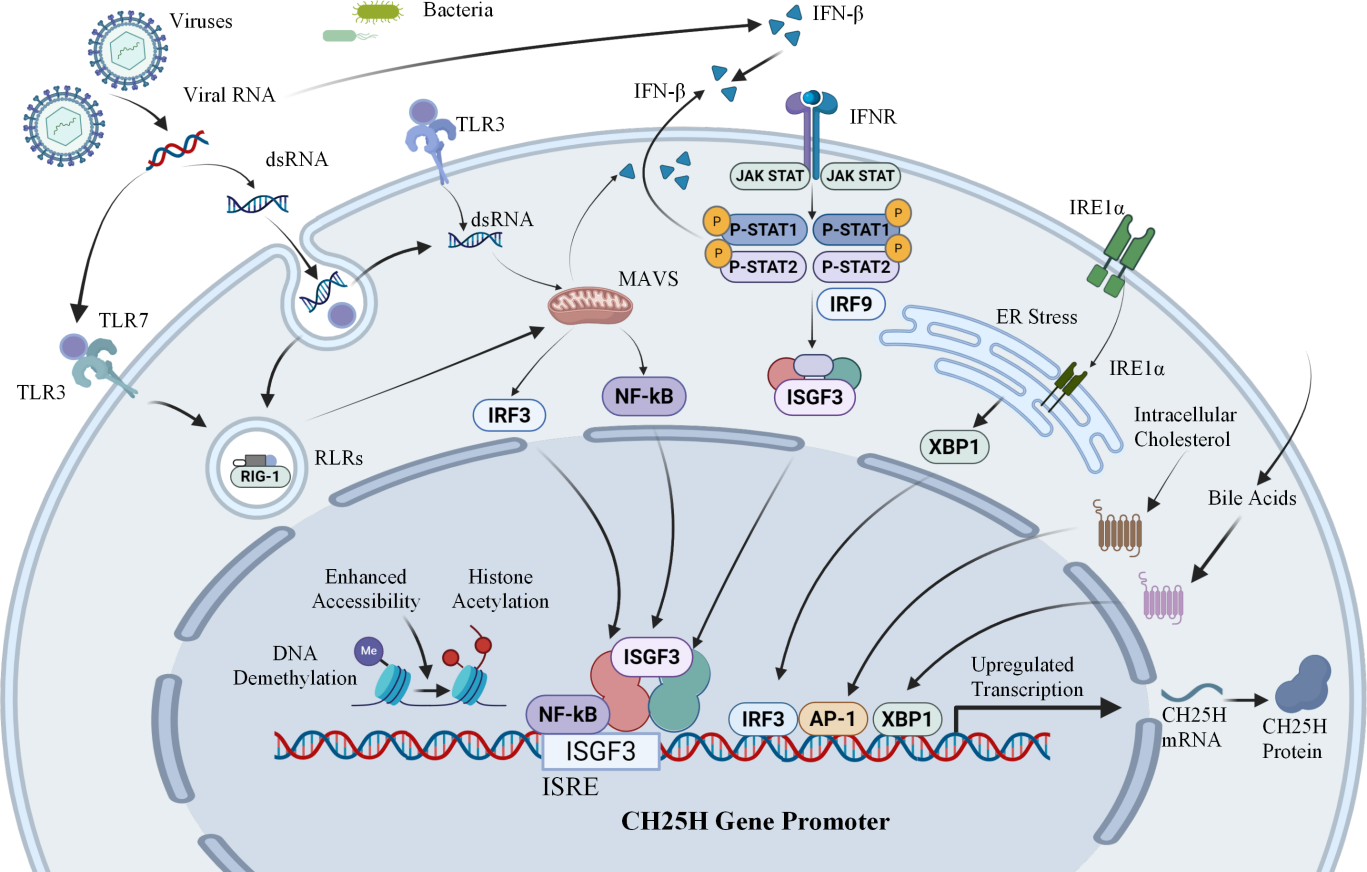
Induced expression of CH25H during viral infection. Upon infection with specific RNA viruses, host cells induce the expression of CH25H through a precise, rapid, and multi-layered regulatory network. This process primarily involves pathogen recognition, signal transduction, and transcriptional activation, and is centered around two main pathways: an interferon (IFN)-dependent pathway and an IFN-independent pathway. In the IFN-dependent pathway, host cells recognize invading RNA viruses—including influenza A virus, vesicular stomatitis virus, respiratory syncytial virus, HIV, and dengue virus—via pattern recognition receptors (PRRs) such as TLR3 and TLR7 on the cell membrane. This recognition activates downstream signaling cascades, inducing the production of type I interferons (IFN-α/β). Secreted IFN-β binds to the interferon receptor (IFNR) on the cell surface, activating the JAK-STAT signaling pathway and leading to the phosphorylation of STAT1 and STAT2. Phosphorylated STAT1/STAT2 form the interferon-stimulated gene factor 3 (ISGF3) complex together with interferon regulatory factor 9 (IRF9). This complex translocates into the nucleus and binds to the interferon-stimulated response element (ISRE) in the promoter regions of target genes, thereby specifically initiating the transcription of numerous antiviral genes, including CH25H. In the IFN-independent pathway, viral nucleic acids generated during infection by viruses such as influenza A virus, influenza B virus, and Ebola virus are directly recognized by cytoplasmic PRRs such as RIG-I. This signal is transduced through the mitochondrial antiviral signaling protein (MAVS) and transforming growth factor-β-activated kinase 1 (TAK1), activating members of the mitogen-activated protein kinase (MAPK) family (ERK, p38, JNK). The activated MAPK pathways further activate downstream key transcription factors, which translocate into the nucleus, bind to the corresponding sites in the promoter region of the CH25H gene, and directly drive its transcription. This enables rapid, early induction of CH25H prior to, or independently of, IFN production. Together, these two pathways ensure that host cells rapidly produce sufficient amounts of 25HC at the early stage of viral infection, which subsequently inhibits viral replication through multiple mechanisms, effectively curtailing the infection. Image created with BioRender.com, with permission.

In addition to the interferon-dependent pathway, viral infection also upregulates CH25H expression via interferon-independent mechanisms (Magoro et al., 2019). Upon binding to RNA ligands generated during viral replication, such as 5′-triphosphate single-stranded RNA or double-stranded RNA, the RIG-I protein undergoes a conformational change from a closed to an open state, driven by recognition via its C-terminal domain (CTD)/repressor domain (RD) and ATP binding within the helicase domain. This conformational shift exposes the N-terminal caspase-recruiting domain (CARD) (Saito et al., 2007; Saito et al., 2008). The activated CARD then interacts with the CARD of the adaptor protein interferon promoter stimulator 1 (IPS-1), located on the outer mitochondrial membrane, thereby initiating downstream signaling (Seth et al., 2005). RIG-I recognizes a broad range of viruses, including Influenza A virus (Kato et al., 2006), Influenza B virus (Loo et al., 2008), and Ebola virus (Habjan et al., 2008), among others. Double-stranded RNA (dsRNA) generated during viral replication can be directly recognized by PRRs, thereby activating the RIG-I-mitochondrial antiviral signaling protein (MAVS), transforming growth factor-β-activated kinase 1 (TAK-1), and mitogen-activated protein kinases (MAPK/ERK/P38/JNK). This cascade subsequently leads to the activation of NF-κB and AP-1, inducing the production of interleukin-8 (IL-8) (Kawai et al., 2005; Takeuchi and Akira, 2009). The regulation of CH25H expression during acute viral infection is illustrated in **Figure 2**.

## 3 Role of CH25H in antiviral response

Accumulating evidence indicates that viruses require cholesterol for entry into host organisms, and cholesterol homeostasis is frequently disrupted during viral invasion (Sun et al., 2011; Stoeck et al., 2018). 25HC can integrate into cellular membranes, altering the stability and integrity of cholesterol-rich membrane domains and thereby inhibiting virus-host cell membrane fusion (Bielska et al., 2014). Following viral infection, CH25H, primarily induced by interferon signaling, demonstrates significant potential in antiviral defense (Blanc et al., 2013). As a key regulator of the sterol biosynthesis negative feedback loop, 25HC exerts its effects mainly by binding to INSIG proteins, thereby inhibiting the activation and processing of SREBP2 and subsequently suppressing cholesterol synthesis (Yan et al., 2021). Lipid depletion activates SREBPs, which markedly enhances the antiviral capacity of 25HC (Blanc et al., 2013). Conversely, antagonizing SREBPs can impede infection by viruses such as hepatitis C virus (HCV), and Zika virus (ZIKV) (Wu et al., 2018a; Singh et al., 2024). 25HC modulates inflammatory responses via the SREBP pathway, suppressing the production of IL-1 family cytokines in macrophages (Reboldi et al., 2014). Notably, SREBP2 itself can directly activate the NLRP3 inflammasome in endothelial cells to promote inflammation (Xiao et al., 2013), suggesting that the SREBP pathway serves as a critical node linking cholesterol metabolism, antiviral immunity, and inflammatory regulation. LXRs, as sterol-sensitive transcription factors activated by 25HC, upregulate the expression of cholesterol-related genes such as ABCA1 and ABCG1 (Kemmerer et al., 2016) and negatively regulate Niemann-Pick C1 like 1 (NPC1L1) (Duval et al., 2006). In the context of antiviral defense, 25HC induces the expression of its own synthase, CH25H, and enhances IFN-β production in an LXR-dependent manner, thereby establishing a positive feedback loop that amplifies the antiviral response (Liu et al., 2018a; Liu et al., 2018b). Cholesterol esterification and its product, lipid droplets (LDs), play pivotal roles in antiviral responses. In PDCoV infection, 25HC treatment induces substantial LD formation, concomitant with upregulated IFN-β mRNA and reduced viral replication (Zhang et al., 2022). Multiple studies have further demonstrated that 25HC stimulates LD biogenesis (Abrams et al., 2020; Wang et al., 2020). These findings collectively indicate that ACAT-mediated LD formation constitutes a key mechanism underlying the antiviral activity of 25HC.

The antiviral effect of 25HC is closely associated with the modulation of membrane cholesterol homeostasis. In a spring viremia of carp virus (SVCV) infection model, 25HC, similar to the zebrafish C-reactive protein (CRP), exhibits antiviral activity potentially by disturbing membrane cholesterol balance, which affects intracellular ROS levels and lysosomal pH, thereby inhibiting viral entry (Bello-Perez et al., 2020). Additionally, 25HC induces the expression of RIG-I and downstream genes in macrophages and endothelial cells, suggesting a potential regulatory role in autophagy (Wang et al., 2012). Importantly, the antiviral activity of cholesterol-modulating pathways is not confined to the plasma membrane. Endolysosomal compartments constitute a major intracellular interface at which cholesterol trafficking, vesicular maturation, acidification, viral fusion, and genome release converge. Late endosomal cholesterol accumulation restricts viral replication and impairs endosomal escape of incoming virions in IAV-infected cell models (Musiol et al., 2013; Kuhnl et al., 2018). Similarly, during human rotavirus infection, 25HC and 27HC disrupt OSBP-VAPA-mediated cholesterol recycling, causing viral particles to be sequestered in late endosomes and preventing their release into the cytoplasm (Civra et al., 2018). Moreover, endosomal cholesterol homeostasis and NPC1-dependent lipid transport have also been linked to RNA replication or cytoplasmic penetration in the context of HCV and reovirus infections, indicating that this pathway can influence multiple stages of the viral life cycle (Stoeck et al., 2018; Ortega-Gonzalez et al., 2022). These findings suggest that CH25H/25HC-mediated regulation of cholesterol homeostasis should be considered part of a broader intracellular cholesterol trafficking network involving the plasma membrane, ER, endosomes, lysosomes, and lipid droplets.

25HC exerts its antiviral effects through both the modulation of inflammatory responses and direct inhibition of viral replication. 25HC induces the expression of ISGs and inflammatory cytokines, including IL-8 and IL-1α, thereby activating inflammatory signaling pathways during viral infection (Serquiña et al., 2021). 25HC alleviates ZIKV-induced inflammatory responses and cell death while reducing viral load in the U-87 MG glioblastoma cell line (Tricarico et al., 2019). Furthermore, by suppressing SREBP2-mediated inflammasome activation, 25HC mitigates inflammation-driven tissue damage, indirectly contributing to antiviral immune function (Reboldi et al., 2014). In an *in vitro* model of PDCoV infection, 25HC reversed virus-induced TGF-β signaling, and experiments with TGF-β inhibitors revealed that TGF-β regulates cholesterol metabolism-related genes, implying that 25HC may influence viral replication by modulating the TGF-β pathway (Zhang et al., 2022). Thus, TGF-β likely represents a target exploited during viral invasion, with 25HC exerting antiviral effects through interference with this pathway. 25HC also impairs viral replication by disrupting post-translational modifications of viral proteins. Specifically, it inhibits the N-glycan maturation of the Lassa virus (LASV) G1 glycoprotein, reducing the infectivity of progeny virions (Shrivastava-Ranjan et al., 2016). This suggests that 25HC curbs viral proliferation by interfering with the glycosylation of viral glycoproteins, thereby suppressing the production of infectious viral particles. Additionally, CH25H restricts porcine reproductive and respiratory syndrome virus (PRRSV) replication by promoting the degradation of nonstructural protein 1 alpha (nsp1α) via the ubiquitin-proteasome pathway (Song et al., 2017).

**Figure 3:**
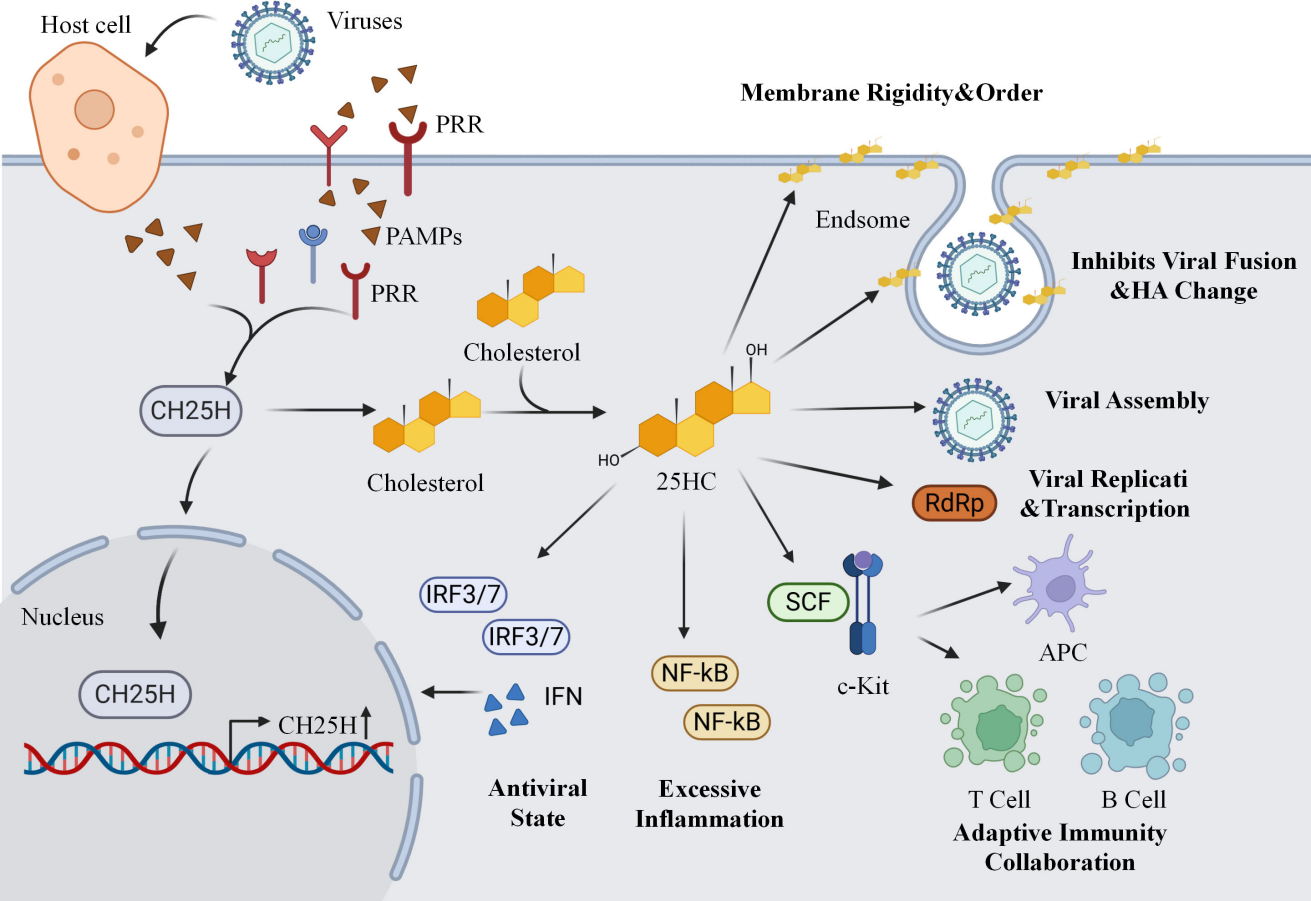
CH25H-mediated antiviral response against RNA viruses. CH25H, via its metabolite 25HC, collaborates with immune pathways such as TLRs, RIG-I, and MAVS. This interaction not only enhances the host’s ability to recognize and clear the virus but also finely regulates the immune response to prevent tissue damage caused by excessive inflammation, providing the host with comprehensive antiviral protection. Image created with BioRender.com, with permission.

In summary, 25HC exerts broad-spectrum antiviral effects through multilayered, multi-pathway mechanisms involving cholesterol metabolism, lipid droplet formation, direct viral inhibition, inflammatory modulation, and interference with viral protein modification. The CH25H-mediated antiviral response is illustrated in **Figure 3**.

## 4 Mechanisms of CH25H against RNA viruses

In the long-term evolutionary arms race between viruses and hosts, hosts have developed a series of complex and sophisticated antiviral immune mechanisms. Among these mechanisms, CH25H and its enzymatic product, 25HC, play a pivotal role in defending against RNA virus infections. They not only modulate intracellular lipid metabolism and alter the composition and fluidity of cellular membranes but also interfere with multiple stages of the viral infection cycle (**Table 1**). The specific mechanisms by which CH25H counteracts different RNA viruses are summarized below.

**Table 1:** Antiviral mechanisms of CH25H against RNA viruses.

Virus	Antiviral mechanism	References
BPIV3	25HC prevents the synthesis of viral antigenomic RNA (cRNA) and genomic RNA (gRNA) in MDBK cells.	(Lv et al., 2019)
DENV	25HC disrupts the fusion of DENV envelope protein with the host membrane by altering cholesterol distribution and lipid raft structure; simultaneously, it inhibits cholesterol biosynthesis required for viral replication, promotes lipid droplet formation and stress response pathways, and initiates innate immune responses.	(Chakraborty et al., 2025)
EBOV	25HC blocks the membrane fusion process between viruses and cells.	(Liu et al., 2013; Wu et al., 2015)
EMCV	25HC inhibits EMCV infection by suppressing viral entry. CH25H interacts with and promotes the degradation of the EMCV RdRp 3D protein to suppress viral replication.	(Li et al., 2020)
JEV	25HC reduces the RNA and protein expression of the JEV envelope protein, modulates cholesterol levels in the host cell membrane, and interferes with viral entry and membrane fusion.	(Archana et al., 2025)
Hantavirus	By inhibiting the activity of 3-hydroxy-3-methyl-glutaryl coenzyme A reductase (HMG-CoA reductase, HMGCR), cholesterol synthesis is reduced.	(Dang et al., 2024)
HCV	25HC inhibits viral membrane fusion and suppresses RNA replication.	(Chen et al., 2014)
HIV	25HC inhibits viral membrane fusion via membrane modification. 25HC restricts HIV entry into host cells by promoting cholesterol efflux and modulating host lipid metabolism.	(Gomes et al., 2018; Saulle et al., 2020)
HRhV	25HC binds to OSBP and disrupts OSBP-mediated cholesterol and PI4P shuttling at membrane contact sites between the ER and Golgi apparatus.	(Roulin et al., 2014)
HRoV	25HC prevents the formation of the OSBP-VAPA complex, thereby interfering with cholesterol recycling between the ER and LEs. This leads to cholesterol accumulation in LEs and subsequent viral sequestration in late endosomal compartments, effectively blocking viral release into the cytoplasm.	(Civra et al., 2018)
Influenza virus	25HC modulates transcriptional responses by amplifying inflammatory signaling via the AP-1 pathway to enhance antiviral immunity.	(Gold et al., 2014)
Lassa virus	CH25H and 25HC interfere with the glycosylation of viral glycoproteins, thereby suppressing viral proliferation	(Shrivastava-Ranjan et al., 2016)
MRV	25HC inhibits the entry of the viral genome into the cytoplasm by altering cell membrane properties, blocking the transport of MRV virus particles to LEs, and delaying the hydrolysis of viral outer membrane proteins and capsid uncoating. 25HC reduces the co-localization of MRV particles with the late endosomal marker protein Rab7 and delays acid-dependent cleavage of the outer capsid protein, resulting in the inability of the virus to efficiently release its genome and thereby reducing infection efficiency.	(Doms et al., 2018)
NDV	25HC inhibits NDV replication by disrupting viral structure and blocking viral entry into cells.	(Nandy et al., 2025; Zhu et al., 2025)
PDCoV	25HC inhibits viral infection by blocking the invasive phase of PDCoV.	(Ke et al., 2021)
PEDV	25HC exhibits broad-spectrum antiviral activity against various PEDV strains by blocking viral entry into host cells.	(Zhang et al., 2019a)
PRRSV	25HC suppresses viral penetration into host cells. CH25H interacts with PRRSV nsp1α and restricts PRRSV replication via nsp1α degradation.	(Song et al., 2017)
RABV	25HC inhibits viral entry into host cells via membrane modification.	(Yuan et al., 2019)
Avian reovirus	25HC inhibits viral entry by delaying the uncoating kinetics of Avian reovirus (ARV), and CH25H blocks p10 protein-induced cell–cell membrane fusion of ARV.	(Wang et al., 2023)
SARS-CoV-2	25HC alters the lipid composition of the cell membrane, directly blocking the membrane fusion process required for viral entry.	(Wang et al., 2020)
SADS-CoV	25HC blocks the membrane fusion process between the virus and the host cell, thereby inhibiting viral replication.	(Liu et al., 2023)
SVA	CH25H suppresses SVA replication. 25HC exerts anti-SVA effects by inhibiting both viral attachment to host cells and replication. The hydroxylase-deficient CH25H mutant (CH25H-M) selectively interacts with SVA 3A protein and promotes its degradation via the ubiquitin-proteasome pathway.	(Zhu et al., 2021)
SVV	25HC blocks viral adsorption to host cells.	(Li et al., 2021)
TGEV	CH25H and 25HC inhibit TGEV replication.	(Zhang et al., 2019a)
ZIKV	25HC inhibits viral entry into host cells via membrane modification.	(Li et al., 2017)

BPIV3, Bovine Parainfluenza Virus Type 3; DENV, Dengue virus; EBOV, Ebola Virus; EMCV, Encephalomyocarditis Virus; JEV, Japanese Encephalitis Virus; HCV, Hepatitis C Virus; HIV, Human Immunodeficiency Virus; HRhV, Human Rhinovirus; HRoV, Human Rotavirus; MRV, Mammalian Orthoreovirus; NDV, Newcastle Disease Virus; PDCoV, Porcine Deltacoronavirus; PEDV, Porcine Epidemic Diarrhea Virus; PRRSV, Porcine Reproductive and Respiratory Syndrome Virus; RABV, Rabies Virus; SARS-CoV-2, Severe Acute Respiratory Syndrome Coronavirus 2; SADS-CoV, Swine Acute Diarrhea Syndrome Coronavirus; SVA, Senecavirus A; SVV, Seneca Valley Virus; TGEV, Transmissible Gastroenteritis Virus; ZIKV, Zika Virus.

### 4.1 Mechanisms of CH25H against RNA viruses in human pathogens

In human viral infections, CH25H and 25HC suppress RNA virus infection and transmission via multiple mechanisms, primarily including the following four aspects:

#### 4.1.1 Inhibition of viral entry and membrane fusion

25HC evidently suppresses the entry and membrane fusion processes of various RNA viruses by modulating cholesterol composition and cellular membrane fluidity. In lung epithelial cells and human lung organoids, 25HC inhibits infection by coronaviruses, including SARS-CoV-2. The underlying mechanism involves the activation of ER-localized acyl-CoA:cholesterol acyltransferase (ACAT) by 25HC, which depletes accessible cholesterol from the plasma membrane, thereby suppressing virus-cell membrane fusion (Wang et al., 2020). For instance, during human rhinovirus (HRhV) infection, 25HC binds to oxysterol-binding protein (OSBP) and disrupts OSBP-mediated cholesterol and phosphatidylinositol 4-phosphate (PI4P) shuttling at membrane contact sites between the ER and Golgi apparatus (Roulin et al., 2014). In human rotavirus (HRoV) infection, 25HC prevents the formation of the oxysterol binding protein (OSBP) -vesicle-associated membrane protein-associated protein-A(VAPA) complex, thereby interfering with cholesterol recycling between the ER and late endosomes (LEs). This leads to cholesterol accumulation in LEs and subsequent viral sequestration in late endosomal compartments, thereby effectively blocking viral release into the cytoplasm (Civra et al., 2018). In human immunodeficiency virus (HIV) infection, 25HC restricts viral entry into host cells by promoting cholesterol efflux and regulating host lipid metabolism (Gomes et al., 2018; Saulle et al., 2020). Similarly, 25HC inhibits membrane fusion of influenza virus, rabies virus (RABV), and ZIKV via membrane modification (Gold et al., 2014; Li et al., 2017; Yuan et al., 2019). These findings demonstrate that 25HC effectively disrupts virus-host cell interactions by altering the physical properties of cellular membranes.

#### 4.1.2 Disruption of viral replication and protein function

CH25H and 25HC further interfere with viral replication via direct interactions with viral proteins. In EMCV infection, CH25H interacts with and promotes the degradation of the viral RNA-dependent RNA polymerase (RdRp 3D) protein, thereby suppressing viral replication (Li et al., 2020). During HCV infection, CH25H binds to the viral non-structural protein 5A (NS5A) protein and inhibits its dimerization, consequently impairing viral replication (Chen et al., 2014). Moreover, 25HC and CH25H effectively inhibit HCV infection by suppressing SREBPs, the key transcription factors that regulate lipid biosynthesis (Xiang et al., 2015). These findings collectively demonstrate that CH25H and 25HC can directly suppress viral replication via multiple distinct mechanisms.

#### 4.1.3 Activation of antiviral immune responses

CH25H and 25HC further suppress viral infection by enhancing the host antiviral immune response. In influenza virus infection, 25HC modulates transcriptional responses by amplifying inflammatory signaling via the AP-1 pathway, consequently promoting antiviral immunity (Gold et al., 2014). These observations demonstrate that CH25H and 25HC directly inhibit viral infection and also reinforce antiviral efficacy via immune system activation.

#### 4.1.4 Inhibition of viral release and transmission

CH25H and 25HC further suppress viral infection by interfering with viral egress and dissemination. In RABV infection, 25HC blocks viral entry into host cells via membrane modification (Yuan et al., 2019). During reovirus infection, 25HC alters the trafficking of viral particles to late endosomes and delays viral uncoating, thereby impeding viral entry into host cells (Doms et al., 2018; Wang et al., 2023). These findings reveal that CH25H and 25HC can effectively restrict viral spread by disrupting intracellular viral transport and release processes.

### 4.2 Antiviral mechanisms of CH25H against RNA viruses in animal pathogens

CH25H and 25HC also play crucial roles in combating RNA virus infections in animal systems. These antiviral mechanisms encompass both direct viral inhibition and indirect modulation of host cellular processes, including the following four aspects:

#### 4.2.1 Inhibition of viral entry and replication

In animal viruses, CH25H and 25HC employ multiple mechanisms to suppress viral entry and replication. CH25H exhibits significant antiviral activity against porcine epidemic diarrhea virus (PEDV), and its hydroxylase-deficient mutant (CH25H-M) retains partial antiviral activity. 25HC exhibits broad-spectrum antiviral activity against diverse PEDV strains by blocking viral entry into host cells. Additionally, both CH25H and 25HC effectively inhibit the replication of transmissible gastroenteritis virus (TGEV) (Zhang et al., 2019a). In porcine reproductive and respiratory syndrome virus (PRRSV) infection, 25HC impedes viral penetration, while CH25H interacts with the viral nonstructural protein 1α (nsp1α) and restricts viral replication via nsp1α degradation (Song et al., 2017). During infection with swine acute diarrhea syndrome coronavirus (SADS-CoV), the expression of host CH25H is upregulated. CH25H and its enzymatic product, 25HC, effectively inhibit SADS-CoV replication by blocking virus-host cell membrane fusion (Liu et al., 2023). In Vero cells, 25HC suppresses Japanese encephalitis virus (JEV) infection by reducing the RNA and protein expression of the viral envelope protein, modulating cholesterol levels in the host cell membrane, and interfering with viral entry and membrane fusion (Archana et al., 2025). Overexpression of CH25H inhibits bovine parainfluenza virus type 3 (BPIV3) replication, and 25HC significantly suppresses BPIV3 infection by preventing the synthesis of viral antigenomic RNA (cRNA) and genomic RNA (gRNA) in MDBK cells (Lv et al., 2019). Newcastle disease virus (NDV) infection induces the upregulation of CH25H in chicken embryo fibroblast cells; overexpression of CH25H inhibits NDV infection, whereas knockdown of its expression promotes viral replication. Furthermore, 25HC markedly suppresses NDV replication by disrupting viral structure and blocking viral entry into cells (Nandy et al., 2025; Zhu et al., 2025). These findings demonstrate that CH25H and 25HC can effectively inhibit viral infection by directly targeting viral entry and replication processes.

#### 4.2.2 Disruption of viral protein function

CH25H and 25HC further inhibit animal viral infections by directly interfering with viral protein function. For instance, in Seneca Valley virus (SVV) infection, 25HC blocks viral adsorption to host cells (Li et al., 2021). In TGEV infection, both CH25H and 25HC suppress viral replication (Zhang et al., 2019a). CH25H exerts its antiviral activity by interacting with the viral nucleoprotein (NP) and reducing the activity of the viral ribonucleoprotein (RNP) complex (Zhu et al., 2025). 25HC, produced by CH25H, inhibits viral entry by delaying the kinetics of Avian reovirus (ARV) uncoating. Additionally, CH25H blocks p10 protein-induced cell–cell membrane fusion mediated by ARV (Wang et al., 2023). These mechanisms indicate that CH25H and 25HC can inhibit viral infection by disrupting critical viral protein functions.

#### 4.2.3 Modulation of host lipid metabolism

CH25H and 25HC also establish an antiviral state by regulating host lipid metabolism during animal viral infections. Hantavirus infection induces CH25H expression in host cells, and either CH25H overexpression or exogenous addition of 25HC significantly inhibits viral infection, likely by suppressing the activity of 3-hydroxy-3-methyl-glutaryl coenzyme A reductase (HMG-CoA reductase, HMGCR) and reducing cholesterol synthesis (Dang et al., 2024). These mechanisms reveal that CH25H and 25HC can effectively restrict viral infection via comprehensive modulation of host lipid metabolic pathways.

In summary, CH25H and 25HC play multifaceted and crucial roles in combating RNA virus infections across both human and animal systems. Their antiviral mechanisms encompass inhibition of viral entry, disruption of viral replication, activation of host immune responses, blockade of viral release, and modulation of host lipid metabolism. These findings not only provide fundamental insights into host antiviral defense mechanisms but also offer promising therapeutic targets for developing novel antiviral strategies. Future research should further elucidate the precise molecular mechanisms of CH25H/25HC action against diverse viruses, potentially enabling the rational design of more effective antiviral therapeutics targeting this important host defense pathway.

## 5 Dynamic regulation of ch25h-mediated antiviral response in RNA virus evasion

The antiviral effect of CH25H/25HC depends both on the infection status of the virus and on the specific stage of the viral replication cycle. In the acute phase of viral infection, CH25H expression is typically upregulated (Kamble et al., 2023). This upregulation promotes the production of 25HC, which binds to the G protein-coupled receptor 183 (GPR183) on immune cells. This binding enhances the local immune response, recruits immune cells to the infection site, and thereby improves the host’s ability to clear the virus (Foo et al., 2023). Viruses may suppress CH25H expression by upregulating immunosuppressive cytokines or modifying the immune microenvironment. Viral infection induces the upregulation of immunosuppressive factors, leading to the decline of antiviral immune responses and consequently inhibiting CH25H expression. In the late phase of infection, particularly in chronic viral infections or cases of viral persistence, CH25H expression may further decrease (Liu et al., 2023). In mouse models of influenza A virus (IAV) and SARS-CoV-2 infection, pulmonary CH25H expression is upregulated, promoting the infiltration of monocytes and macrophages into the lungs via the 25HC receptor GPR183 (Foo et al., 2023). Knockout of the *GPR183* gene or treatment with a GPR183 antagonist significantly reduces macrophage accumulation and inflammatory cytokine production in the lungs of infected mice and alleviates the severity and viral load of SARS-CoV-2 infection (Foo et al., 2023). However, in mouse models, exogenous supplementation with 25HC does not inhibit SARS-CoV-2 replication or the associated pathological damage, weight loss, or survival rate; instead, it exacerbates microvascular injury and inflammatory responses (Fessler et al., 2023). Furthermore, inhibition of downstream signaling pathways of 25HC (via the EBI2/GPR183 receptor) or knockout of the relevant genes did not significantly affect viral load or disease progression in mice (Fessler et al., 2023). The downregulation of CH25H expression in the late phase of infection may also further suppress the host antiviral immune response by altering the immune microenvironment.

During the long-term co-evolution between RNA viruses and hosts, RNA viruses have developed various evasion mechanisms to counteract the CH25H-mediated antiviral response (**Table 2**), while the host maintains immune homeostasis via complex feedback regulation. This dynamic interplay between RNA viruses and hosts constitutes the core of virus-host interactions. One well-characterized mechanism is the direct downregulation of CH25H expression at the transcriptional and post-translational levels. For instance, porcine reproductive and respiratory syndrome virus (PRRSV) actively degrades porcine CH25H via the ubiquitin-proteasome pathway through its envelope (E) protein, which directly interacts with CH25H and promotes its ubiquitination at Lys28, thereby attenuating 25HC production and antiviral efficacy (Ke et al., 2019). Similarly, porcine circovirus type 3 (PCV3) infection downregulates CH25H mRNA expression, a process linked to immunosuppression and immune evasion, thereby facilitating viral replication (Zhang et al., 2024a). Beyond direct protein degradation, viruses also exploit host regulatory networks to suppress CH25H. In EV71 and CVB3 infected cells, miR-7705, which is upregulated in a STAT1-dependent manner, directly targets the 3′UTR of CH25H mRNA and negatively regulates its expression, thereby limiting CH25H-mediated restriction of RNA viruses (Wang et al., 2025). Viruses may further interfere with upstream signaling pathways that govern CH25H induction. Many RNA viruses have evolved mechanisms to antagonize the type I interferon (IFN) response by targeting key molecules such as TBK1 (HCV NS2/NS3) (Otsuka et al., 2005; Kaukinen et al., 2013), MAVS (cleaved by HCV NS3/4A) (Meylan et al., 2005) and STING (targeted by HCV NS4B) (Ding et al., 2013; Nitta et al., 2013), thereby suppressing the JAK-STAT pathway and subsequent ISG expression, including CH25H (Xu and Zhong, 2016). Collectively, these multifaceted evasion strategies underscore the complex and dynamic arms race between RNA viruses and host innate immunity.

**Table 2:** Evasion strategies of RNA viruses targeting the CH25H/25HC axis.

Potential escape strategy	Molecular mechanism	Representative RNA viruses	Major infection phase	References
Proteasome-mediated degradation of CH25H	Viral E proteins promote ubiquitin–dependent proteasomal degradation of CH25H, thereby attenuating CH25H-mediated antiviral activity.	PRRSV	Early phase/innate immune evasion	(Ke et al., 2019)
Lysosomal degradation of CH25H	Viral nonstructural proteins (e.g., Nsp1β, Nsp11) promote lysosome-dependent CH25H degradation, suppressing ISG function.	PRRSV	Early infection	(Dong et al., 2018)
Inhibition of IFN-mediated CH25H induction	Viral proteins antagonize the IFN signaling pathway, suppressing transcriptional induction of CH25H as an interferon-stimulated gene (ISG).	HCV	Early phase/innate immune sensing	(Ding et al., 2013; Xu and Zhong, 2016)
Evasion of 25HC-mediated entry restriction	Viruses counteract 25HC-induced alterations in membrane cholesterol distribution and biophysical properties that impair viral attachment, entry, or membrane fusion	HIV, EBOV	Early phase/attachment, entry and membrane fusion	(Liu et al., 2013; Gomes et al., 2018)
Escape from oxysterol-induced endosomal sequestration	Viral particles evade retention in late endosomes induced by oxysterols (e.g., 25HC), potentially via modulation of endosomal trafficking or uncoating	HRoV	Early post-entry phase/endosomal trafficking and uncoating	(Civra et al., 2018)
miRNA-mediated post-transcriptional regulation of CH25H	IFN-induced miRNAs target the CH25H 3′ UTR and suppress CH25H expression, thereby attenuating CH25H-dependent antiviral activity.	EV71, CVB3	Early-to-replication phase/host feedback regulation	(Wang et al., 2025)
Counteraction of CH25H enzyme activity-dependent restriction	CH25H restricts viral replication through both 25HC-dependent enzymatic mechanisms and enzyme activity-independent mechanisms, such as interference with viral replication-associated proteins or replication complex formation. Viruses that remain productive under CH25H pressure may escape, tolerate, or bypass these antiviral restrictions.	HCV, SVA, EMCV	Replication phase/mid-to-late infection	(Chen et al., 2014; Li et al., 2020; Zhu et al., 2021)

In response to RNA viral evasion strategies, the host has also evolved complex feedback regulatory mechanisms to maintain immune balance. During the late phase of viral infection, the host upregulates the expression of CH25H-associated genes to enhance CH25H activity in combating RNA viral evasion. STAT1 induces the expression of CH25H, promoting the synthesis of the antiviral compound 25HC (Matsumiya and Imaizumi, 2013). 25HC can be metabolized to the less active 25HC-3-sulfate, thereby partially diminishing its antiviral activity (Liu et al., 2018b). Furthermore, host cells actively metabolize 25HC to prevent its cytotoxic accumulation. 25HC is converted by the enzyme cytochrome P450 family 7 subfamily B member 1 (CYP7B1) into 7α, 25-dihydroxycholesterol (7α, 25-OHC), effectively reducing intracellular 25HC concentration and forming an important clearance feedback loop (Zhang et al., 2024b). These feedback regulatory mechanisms ensure the dynamic balance of the CH25H/25HC antiviral response, preventing excessive immune damage. Additionally, some RNA viruses encode specific proteins that directly inhibit CH25H enzymatic activity or interfere with CH25H’s subcellular localization. Although CH25H inhibits HCV replication through its product 25HC, a CH25H mutant lacking enzymatic activity still exhibits specific antiviral activity against HCV. CH25H directly interacts with the HCV NS5A protein, inhibiting the formation of dimers necessary for replication, demonstrating that CH25H exerts its antiviral function through both enzyme activity-dependent and -independent (directly targeting viral proteins) pathways (Chen et al., 2014). Similarly, early infection with Avian reovirus (ARV) upregulates the expression of chicken CH25H (chCH25H). CH25H/25HC blocks viral entry by delaying the uncoating process of ARV and also inhibits cell-cell membrane fusion induced by the ARV p10 protein (Wang et al., 2023). In a rhesus macaque model of chronic SIVmac239 infection, compared with antiretroviral therapy (ART) alone, 25HC combined with ART effectively controlled SIV replication, enhanced SIV-specific cellular immune responses, restored the CD4/CD8 ratio, reversed the hyperactivation state of CD4^+^ T cells, and inhibited the secretion of pro-inflammatory cytokines by CD4+ and CD8+ T lymphocytes (Wu et al., 2021). The host regulates the CH25H/25HC axis to maintain immune homeostasis, exerting antiviral effects via enzymatic (25HC) and non-enzymatic (protein-targeting) mechanisms. Future work should harness this dual pathway as a broad-spectrum strategy against emerging RNA viruses.

The dynamic balance between RNA viral evasion and host feedback regulation reveals the complexity of RNA virus-host interactions and concurrently provides important insights for developing novel antiviral therapies targeting CH25H regulation. Both targeting the regulatory pathways of CH25H to enhance CH25H’s antiviral effects and inhibiting RNA viral evasion mechanisms to restore CH25H’s antiviral activity are potential therapeutic strategies. Additionally, regulating the host cell cholesterol metabolic pathway to maintain physiological concentrations of 25HC can also effectively inhibit RNA viral replication and spread. In conclusion, a deeper understanding of the dynamic balance between RNA virus evasion and CH25H feedback regulation will help elucidate the molecular mechanisms of RNA virus-host interactions and concurrently provide important theoretical foundations for developing novel antiviral therapies.

## 6 Endolysosomal cholesterol homeostasis and RNA virus infection

Mounting evidence indicates that endolysosomal cholesterol homeostasis represents an important host-dependent vulnerability shared by multiple RNA viruses. Unlike virus-encoded proteins, host lipid-trafficking machineries are relatively conserved and are required by diverse viruses to complete entry, fusion, uncoating, cytoplasmic penetration, or early replication events. Endolysosomes are not merely degradative organelles but also dynamic sorting hubs that regulate membrane composition, vesicular maturation, luminal acidification, pH-dependent proteolysis, and cholesterol transport (Glitscher and Hildt, 2021; Post et al., 2025). These host-cell processes are exploited by many emerging viruses to establish productive infection, while pharmacological disruption of endolysosomal cholesterol homeostasis may provide a rational host-directed target with potential broad-spectrum activity (Kaufmann et al., 2018; Kummer et al., 2022).

### 6.1 Endolysosomal cholesterol homeostasis as a host-directed antiviral target

Mechanistic evidence from different viral infections demonstrates that endolysosomal cholesterol homeostasis plays significant roles in multiple steps of viral infection. Taking IAV as an example, annexin A6-balanced late endosomal cholesterol controls viral replication and propagation, while experimentally induced late endosomal/lysosomal cholesterol accumulation impairs viral endosomal escape by reducing viral-endosomal membrane mixing (Musiol et al., 2013; Kuhnl et al., 2018). Likewise, in HRoV infection, 25HC and 27HC disrupt OSBP and VAPA-mediated cholesterol recycling between the endoplasmic reticulum and late endosomes, leading to viral sequestration in late endosomal compartments and preventing cytoplasmic release (Civra et al., 2018). For HCV infection, viral RNA replication depends on functional lipid transport along the endosomal-lysosomal pathway, indicating that endosomal cholesterol homeostasis can also support replication-complex formation rather than only viral entry (Stoeck et al., 2018). Additionally, NPC1-mediated endosomal cholesterol homeostasis regulates reovirus penetration into the cytoplasm, whereas NPC1 is required for EBOV glycoprotein-dependent entry and escape from endosomal compartments (Carette et al., 2011; Ortega-Gonzalez et al., 2022). Taken together, these findings indicate that endolysosomal cholesterol trafficking can influence multiple stages of the viral life cycle, including entry, membrane fusion, uncoating, genome delivery, and replication.

Pharmacological studies further suggest that this host-cell process is therapeutically targetable. The clinically licensed antifungal drug itraconazole has been shown to inhibit IAV infection *in vitro* and *in vivo*, with proposed mechanisms involving perturbation of cellular cholesterol balance and endolysosomal cholesterol trafficking (Schloer et al., 2019). In SARS-CoV-2 infection, fluoxetine and other functional inhibitors of acid sphingomyelinase impaired infection by targeting the endolysosomal host-virus interface, with mechanistic evidence indicating altered endolysosomal acidification and cholesterol accumulation (Schloer et al., 2020a). Similarly, itraconazole- or fluoxetine-induced endolysosomal cholesterol imbalance impaired EBOV infection *in vitro*, supporting the idea that endolysosomal cholesterol transport is a host-cell process required by phylogenetically distinct RNA viruses (Kummer et al., 2022). Together, these studies provide a rationale for considering endolysosomal cholesterol homeostasis as a druggable antiviral interface rather than as a virus-specific phenomenon. Moreover, 3D ex vivo tissue platforms provide physiologically relevant models for investigating the early phases of IAV- and SARS-CoV-2-induced respiratory infection and may facilitate the evaluation of host-directed interventions in tissue contexts closer to the human airway (Schloer et al., 2022).

These findings are mechanistically relevant to the CH25H/25HC axis. CH25H-derived 25HC reshapes cholesterol distribution by inhibiting SREBP-dependent cholesterol synthesis, activating LXR-dependent cholesterol efflux programs, promoting cholesterol esterification, and altering membrane cholesterol accessibility (Wang et al., 2020; Zhao et al., 2020). Although CH25H/25HC and itraconazole/fluoxetine/FIASMAs act through partially distinct molecular targets, they converge functionally on the regulation of intracellular cholesterol availability, vesicular cholesterol trafficking, and membrane-dependent viral entry. Thus, the CH25H/25HC axis involved in RNA virus infection should not be considered in isolation, but rather as one component of a broader cholesterol homeostatic network that includes endolysosomal cholesterol transporters, acid sphingomyelinase activity, lipid droplet formation, and sterol-sensitive transcriptional programs (Fessler, 2016; Mathiowetz and Olzmann, 2024).

### 6.2 Combination strategies integrating cholesterol-modulating host-directed drugs with direct-acting antivirals

Combination therapy provides a rational translational route for exploiting cholesterol-centered host restriction while preserving the virus-specific potency of direct-acting antivirals (DAAs). This strategy is conceptually supported by the broader field of host-directed antiviral therapy, in which targeting conserved host-cell processes may complement virus-directed inhibition and reduce the likelihood that resistance emerges through mutation of a single viral target (Kaufmann et al., 2018; Kumar et al., 2020). In influenza and other RNA virus infections, combination antiviral strategies have also been proposed to improve antiviral potency, broaden coverage, and reduce the selection of drug-resistant variants, although their efficacy must be evaluated in virus- and model-specific contexts (Dunning et al., 2014; Koszalka et al., 2022). DAAs such as remdesivir, its parent nucleoside GS-441524, or oseltamivir inhibit defined viral enzymes or replication steps: remdesivir targets the coronavirus RNA-dependent RNA polymerase, whereas oseltamivir inhibits influenza neuraminidase and thereby interferes with progeny virion release (Moscona, 2005; Gordon et al., 2020; Kokic et al., 2021). In contrast, host-directed drugs such as itraconazole, fluoxetine, and other FIASMAs perturb cellular or endolysosomal cholesterol homeostasis, endosomal trafficking, and virus-cell entry interfaces (Schloer et al., 2020a; Glitscher and Hildt, 2021; Kummer et al., 2022). Because these two drug classes act on mechanistically distinct targets, their combination may lower the effective concentration of individual drugs, expand the antiviral window, and reduce the probability that a single viral mutation will confer high-level resistance.

Experimental evidence supports this concept, although the interaction pattern differs among viruses, cell systems, and drug pairs. In SARS-CoV-2 infection, combined treatment with remdesivir and the repurposed host-directed drugs fluoxetine or itraconazole synergistically impaired viral infection *in vitro*, indicating that targeting the viral polymerase and the host endolysosomal interface can produce greater antiviral efficacy than either approach alone (Schloer et al., 2021). A related study showed that fluoxetine combined with GS-441524 was well tolerated in a polarized Calu-3 cell culture model and exerted synergistic antiviral effects against different SARS-CoV-2 variants, strengthening the rationale for pairing nucleoside analogues with endolysosome-targeting host-directed drugs (Brunotte et al., 2021). For IAV, the combination of oseltamivir and itraconazole produced stronger antiviral activity than oseltamivir monotherapy in polarized bronchial epithelial Calu-3 cells and enabled lower oseltamivir concentrations; however, drug-interaction analyses indicated predominantly additive effects with limited condition-dependent synergy rather than uniformly strong synergy (Schloer et al., 2020b). This distinction is important because it prevents overinterpretation while still supporting combination therapy as a strategy to increase antiviral robustness.

The CH25H/25HC axis provides a conceptual bridge between endogenous antiviral immunity and these pharmacological approaches. Both 25HC and cholesterol-modulating drugs can alter accessible cholesterol pools and membrane trafficking, whereas DAAs suppress viral replication after entry or interfere with viral egress. Thus, combination strategies could theoretically block infection at multiple stages, including entry, endosomal escape, replication, and spread. Nevertheless, clinical translation requires careful pharmacokinetic and safety evaluation. Itraconazole is widely used as a strong CYP3A inhibitor in clinical drug-drug interaction studies, indicating that its combination with other antiviral agents requires close assessment of metabolic interactions and systemic exposure (Chen et al., 2019). Fluoxetine and other FIASMAs may exert cell-type- and dose-dependent effects on lysosomal function, endolysosomal acidification, and sphingolipid/cholesterol metabolism, which may influence both antiviral efficacy and safety (Schloer et al., 2020a; Loas and Le Corre, 2021). More broadly, excessive disruption of cholesterol trafficking may impair normal lysosomal, immune, or neurological functions because intracellular cholesterol distribution is tightly linked to immune signaling and cellular homeostasis (Fessler, 2016; Glitscher and Hildt, 2021). Future studies should therefore integrate physiologically relevant airway or organoid models, animal infection models, PK/PD-guided dosing, and biomarkers of cholesterol/endolysosomal perturbation to define when host-directed cholesterol modulation is beneficial, neutral, or potentially harmful.

## 7 Limitations of current research and future research directions

Although previous studies have noted that CH25H-mediated antiviral responses play a significant role in host defense against RNA viruses, significant differences exist in how various RNA viruses regulate CH25H expression and function. Current research primarily focuses on human RNA viruses including HIV, HCV, and SARS-CoV-2, while the effects of other RNA viruses (e.g., DENV and EBOV) on CH25H and their regulatory mechanisms remain unclear. This limitation hinders a comprehensive understanding of the universal role of CH25H in antiviral responses. Furthermore, viruses suppress CH25H expression or function via multiple mechanisms; however, the relative importance and synergistic effects of these mechanisms in different viral infections have not been comprehensively investigated. This hampers a comprehensive understanding of viral escape strategies targeting CH25H.

From a translational perspective, targeting endolysosomal cholesterol homeostasis has several potential advantages. First, because it targets host pathways rather than viral proteins, it may impose a higher genetic barrier to viral resistance. Second, because many RNA viruses depend on related endosomal entry, fusion, uncoating, or replication-organelle remodeling processes, this strategy may exert broad-spectrum antiviral effects. Third, the availability of clinically licensed compounds such as itraconazole and fluoxetine provides a foundation for drug repurposing. Nevertheless, these approaches require careful evaluation because endolysosomal cholesterol transport, lysosomal pH, sphingolipid metabolism, and immune signaling are essential for normal cellular physiology. Moreover, excessive or improperly timed manipulation of 25HC-related pathways may aggravate inflammatory injury in certain infection contexts, as suggested by studies showing context-dependent detrimental effects of therapeutic or endogenous 25HC in lung injury and SARS-CoV-2 pathogenesis models (Fessler et al., 2023; Madenspacher et al., 2023). Future studies should therefore define virus-specific therapeutic windows, cell-type-dependent responses, delivery strategies, and safety profiles before translating cholesterol-modulating host-directed therapies into clinical antiviral use.

Future research should also focus on investigating how the host immune system and cellular metabolic states impact CH25H expression and function. It is essential to establish multi-level regulatory models to comprehensively elucidate the dynamic regulatory mechanisms of CH25H-mediated antiviral responses. Furthermore, the interactions between viral proteins and host regulatory factors, the influence of epigenetic modifications, and the aberrant changes in intracellular cholesterol metabolism should be comprehensively analyzed. This approach will provide a theoretical foundation for the development of novel CH25H-targeted antiviral strategies. Furthermore, comprehensive patient profiling, which integrates genetic testing, immunophenotyping, and metabolomic analysis, combined with insights into the dynamic regulatory mechanisms of CH25H, can facilitate the development of personalized treatment regimens for precision medicine. Moreover, multi-omics integration (i.e., genomics, transcriptomics, proteomics, and metabolomics) will enable the identification of novel CH25H-interacting biomolecules and signaling pathways, thereby expanding the repertoire of potential targets for next-generation antiviral strategies. Finally, artificial intelligence (AI)-driven analysis of CH25H-associated gene expression profiles and protein-protein interaction networks during diverse RNA viral infections can precisely identify key CH25H regulatory factors and critical signaling pathways. Machine learning algorithms can further predict viral evasion mechanisms and interindividual variations in CH25H expression patterns, ultimately facilitating the design of patient-specific antiviral therapies with improved efficacy and safety profiles.

Although targeting the CH25H/25HC axis represents an attractive direction for developing novel broad-spectrum antiviral strategies, its clinical translation requires cautious evaluation. Its therapeutic potential lies in the fact that, by upregulating host intrinsic defense or applying 25HC analogues, it might provide broad-spectrum protection against multiple viruses with a high barrier to resistance. However, cholesterol metabolism and the physiological functions of 25HC are highly cell- and tissue-specific; systemic intervention may interfere with normal functions of the immune, nervous, or endocrine systems, and may pose metabolic risks due to disruption of global cholesterol homeostasis. Moreover, the dual role of CH25H in innate immune regulation and cholesterol metabolism implied that enhancing its levels at an inappropriate time might exacerbate pathological immune responses. Therefore, future CH25H/25HC-based translational research should develop precise targeted strategies, conduct personalized assessments based on individual patients’ immune and cholesterol metabolic statuses, and consider its use as part of combination therapy, in order to maximize antiviral efficacy while minimizing potential side effects.

In summary, in-depth investigation into the dynamic regulatory mechanisms of CH25H-mediated antiviral responses during RNA viral immune evasion will not only provide comprehensive insights into host-virus interactions but also establish a critical theoretical foundation and technical framework for developing novel antiviral therapeutics and implementing personalized precision medicine. These advances carry significant scientific implications and substantial clinical translational value.

## 8 Conclusion

CH25H acts as a critical antiviral effector by producing 25HC, which inhibits viral replication, remodels cholesterol homeostasis, and modulates immune responses. However, RNA viruses employ sophisticated evasion strategies, including suppression of CH25H expression, disruption of 25HC-mediated antiviral activity, and co-option of host lipid metabolic pathways. The stage-specific dynamic regulation of CH25H during viral infection, together with its coordination with innate immune signaling and intracellular cholesterol trafficking, establishes a multi-tiered antiviral defense network. Recent studies on endolysosomal cholesterol homeostasis further extend this framework by identifying a conserved host-dependent interface exploited by diverse RNA viruses, including IAV, SARS-CoV-2, and EBOV. Targeting this interface, especially through rational combinations of cholesterol-modulating host-directed drugs and DAAs, may provide a promising strategy to broaden antiviral activity and reduce resistance development. Therefore, deeper investigation of the CH25H/25HC axis and its connection with endolysosomal cholesterol regulation will provide fundamental insights into host-virus interactions and support the development of next-generation antiviral interventions.
